# Growth in representation of Saudi scientists among Stanford's top 2 percent most-cited (2019–2023)

**DOI:** 10.3389/frma.2025.1685185

**Published:** 2025-10-02

**Authors:** Luluah Altukhaifi, Nouf Alturaiki, Khaled Al-hadyan

**Affiliations:** ^1^Research Affairs Department, Research and Innovation, King Faisal Specialist Hospital and Research Centre (KFSHRC), Riyadh, Saudi Arabia; ^2^Radiation Biology Section, Biomedical Physics Department, Research and Innovation, King Faisal Specialist Hospital and Research Centre (KFSHRC), Riyadh, Saudi Arabia; ^3^Research Laboratories Department, Research and Innovation, King Faisal Specialist Hospital and Research Centre (KFSHRC), Riyadh, Saudi Arabia

**Keywords:** Stanford's top 2 percent most-cited scientists, Saudi Arabia, bibliometric analysis, citation metrics, h-index

## Abstract

Global citation-based databases, such as Stanford University's Top 2% Scientists (SUD2%) database, offer powerful tools for tracking high-impact researchers. Despite Saudi Arabia's growing investment in scientific research, a longitudinal analysis of its presence in these elite citation rankings has been lacking. This study provides the first 5-year analysis (2019–2023) of Saudi-affiliated scientists listed in the SUD2% (single-year category), evaluating their growth in numbers, performance indicators, disciplinary distribution, and gender representation. Data were extracted from Elsevier's Mendeley-hosted SUD2% dataset. The key bibliometric metrics under analysis included the average composite citation score (C-score), citation rank, total citations, and h-index. A one-way repeated measures ANOVA on ranks was used to assess statistical differences between Saudi-affiliated and global scientists. Gender classification was performed using NamSor, based on validated confidence thresholds. The number of Saudi-affiliated scientists in the SUD2% nearly tripled from 556 in 2019 to 1,684 in 2023. Significant gains were also observed in average C-scores (*p* = 0.003), citation ranks (*p* = 0.002), total citations (*p* = 0.001), and h-indices (*p* = 0.025). Disciplinary analysis revealed continued dominance in clinical medicine, chemistry, and biomedical research. Gender analysis revealed male dominance (93.9%) over the 5-year period, although female representation increased from 5.0% in 2019 to 7.3% in 2023. Saudi Arabia's scientific community is making statistically significant progress in high-impact research, evidenced by increasing representation and improved citation metrics in global SUD2% rankings. While gaps remain—particularly in gender representation and individual citation ranks—trends point toward sustained momentum and broadening institutional participation in global research excellence.

## 1 Introduction

Scientific productivity and impact are increasingly being measured through citation-based metrics, with several databases and methodologies being developed to identify the most influential researchers globally (Hirsch, 2005; Bornmann and Daniel, 2008; Ioannidis et al., 2019; Clarivate, 2024; Elsevier, 2025). One of the most widely recognized global citation-based rankings is the Stanford University database of the top 2% most-cited scientists (SUD2%), which systematically identifies the most influential researchers worldwide based on standardized citation indicators (Ioannidis et al., 2019; Ioannidis, 2024). The SUD2% offers separate rankings for career-long impact and single-year citation performance, allowing for the nuanced assessment of both historical influence and recent activities. All indicators were presented with and without self-citations. Additional measures, such as the ratio of citations to citing papers, were included to help flag potential citation manipulation or clustering (Ioannidis et al., 2019; Ioannidis, 2024). Scientists listed in the SUD2% were classified into 22 major scientific fields and 176 subfields using the Science-Metrix classification system. Percentile ranks within each field were provided for every researcher who published at least five Scopus-indexed papers (Ioannidis et al., 2019; Ioannidis, 2024). The database utilizes a composite citation indicator that integrates six key metrics: total citations, the Hirsch h-index, the co-authorship-adjusted Schreiber hm-index, citations of single-authored papers, citations of single or first-authored papers, and citations of single, first, or last-authored papers (Ioannidis et al., 2016).

Although some bibliometric studies have positioned Saudi Arabia's research trajectory within broader global and regional contexts (Al-Marzouqi and Arabi, 2022; Ajayan et al., 2022), there is still limited longitudinal evidence assessing Saudi scientists' presence in global citation benchmarks, particularly within selective, individual-level rankings that capture current research impact. To address this gap, the present paper offers a comprehensive assessment of the growth in Saudi-affiliated scientists' representation, performance metrics, and disciplinary trends within the SUD2% (single-year category) over a five-year period from 2019 to 2023.

## 2 Materials and methods

This study quantified the annual inclusion of Saudi-affiliated scientists in the SUD2% and compared their performance against global trends using the following key bibliometric indicators: average composite citation score (c-score), average rank, total citations, and h-index. The disciplinary distribution of Saudi scientists was analyzed to identify any structural patterns across scientific fields and assess any temporal shifts in field-specific prominence. The data were extracted from the SUD2% dataset (single-year impact category), which is publicly available via Elsevier's Mendeley Data repository (Ioannidis et al., 2019; Ioannidis, 2024).

All data extraction was conducted using Microsoft Excel's sorting and filtering functions, with custom parsing to isolate Saudi-affiliated entries per year. The ranking values used in the analysis were based on the actual sorted order of scientists in the Excel sheets, rather than the “rank (ns)” column included in the dataset. This decision was made following the observation of inconsistencies between the two ranking metrics—particularly beyond the top 100,000 scientists—in which the “rank (ns)” field occasionally diverged from the expected sequential order. The manually derived rank from the dataset structure was therefore deemed more accurate and consistently applied throughout the analysis. Only data from the “citations excluding self-citations” category were used to ensure a more accurate, unbiased representation of external scientific recognition, as citation metrics that include self-citations may inflate the perceived impact of a scientist's work.

It is important to note that two versions of the 2021 SUD2% dataset were released. The initial version was published on October 10, 2022, and a subsequent updated version was released on November 3, 2022. The updated version was selected for this study because it was presumed to include corrections or refinements and thus offer greater accuracy.

In a few cases, some scientists were erroneously listed as having Saudi affiliation despite being associated with non-Saudi institutions. Six such instances were identified: one in the 2019 list (University of Newcastle), three in 2020 (United Arab Emirates University, University of Cape Town, and University of Technology Sydney), and two in 2022 (Khalifa University of Science and Technology). Upon review, extensive co-authorship links with Saudi-affiliated researchers were identified, which may explain the misattribution of country affiliation by automated algorithms. Given the small number of cases (6 of 5,593 Saudi-flagged entries, or approximately 0.1%), and to avoid exclusion bias, these individuals were retained in the Saudi-affiliated group for consistency and transparency.

NamSor (https://namsor.app/) was employed to assess gender representation among Saudi-affiliated scientists listed in the SUD2%. This is a name-based gender classification software that predicts gender using onomastic analysis. This software has been previously validated and applied in Stanford's SUD% bibliometric research of Stanford University to analyze the large-scale gender distribution of scientific authorship (Ioannidis et al., 2023). Each name in the SUD% dataset was algorithmically assigned to one of three categories: male, female, or uncertain. The latter was used when the NamSor software predicted the gender with a confidence score below 85%.

All figures and statistical analyses were performed using SigmaPlot version 14.5 for Windows (Systat Software Inc., Chicago, IL, USA). A One-Way Repeated Measures Analysis of Variance on Ranks was applied to evaluate the statistical significance of differences between Saudi-affiliated scientists and global averages across the main bibliometric indicators (c-score, rank, citations, h-index). A *p*-value of < 0.05 was considered statistically significant.

This study received ethical clearance for publication from the Research Affairs Department on June 29, 2025 (no. 2255967).

## 3 Results

### 3.1 Trends in Saudi representation

As presented in Table 1, the number of Saudi-affiliated scientists featured in the SUD2% single-year category has shown consistent, substantial growth from 2019 to 2023. The number of researchers increased from 556 in 2019 to 1,684 in 2023, nearly tripling over 5 years. This expansion increased Saudi Arabia's relative share of global representation in the SUD2% from 0.34% to 0.75%. The raw data of the Saudi-affiliated scientists (2019–2022) is shown in Supplementary Data 1. At the institutional level, the number of Saudi institutions listed in the SUD2% rose from 50 in 2019 to 69 in 2023. While the total number of global institutions remained relatively constant, the proportion of Saudi institutions rose modestly from 0.30% to 0.35%, underscoring a broader growth in institutional research involvement that is making a sustained global impact.

**Table 1:** Saudi representation in Stanford university's top 2%.

**Year**	**Saudi-affiliated scientists**	**Global top 2% scientists**	**%**	**Saudi institutions**	**Global institutions**	**%**
2019	556	161,441	0.34	50	16,481	0.30
2020	870	190,063	0.46	51	19,542	0.26
2021	1,020	200,196	0.51	54	20,438	0.26
2022	1,321	210,198	0.63	67	21,793	0.31
2023	1,684	223,153	0.75	69	19,733	0.35

Single-year category (2019–2023).

### 3.2 Performance indicators

**Figure 1:**
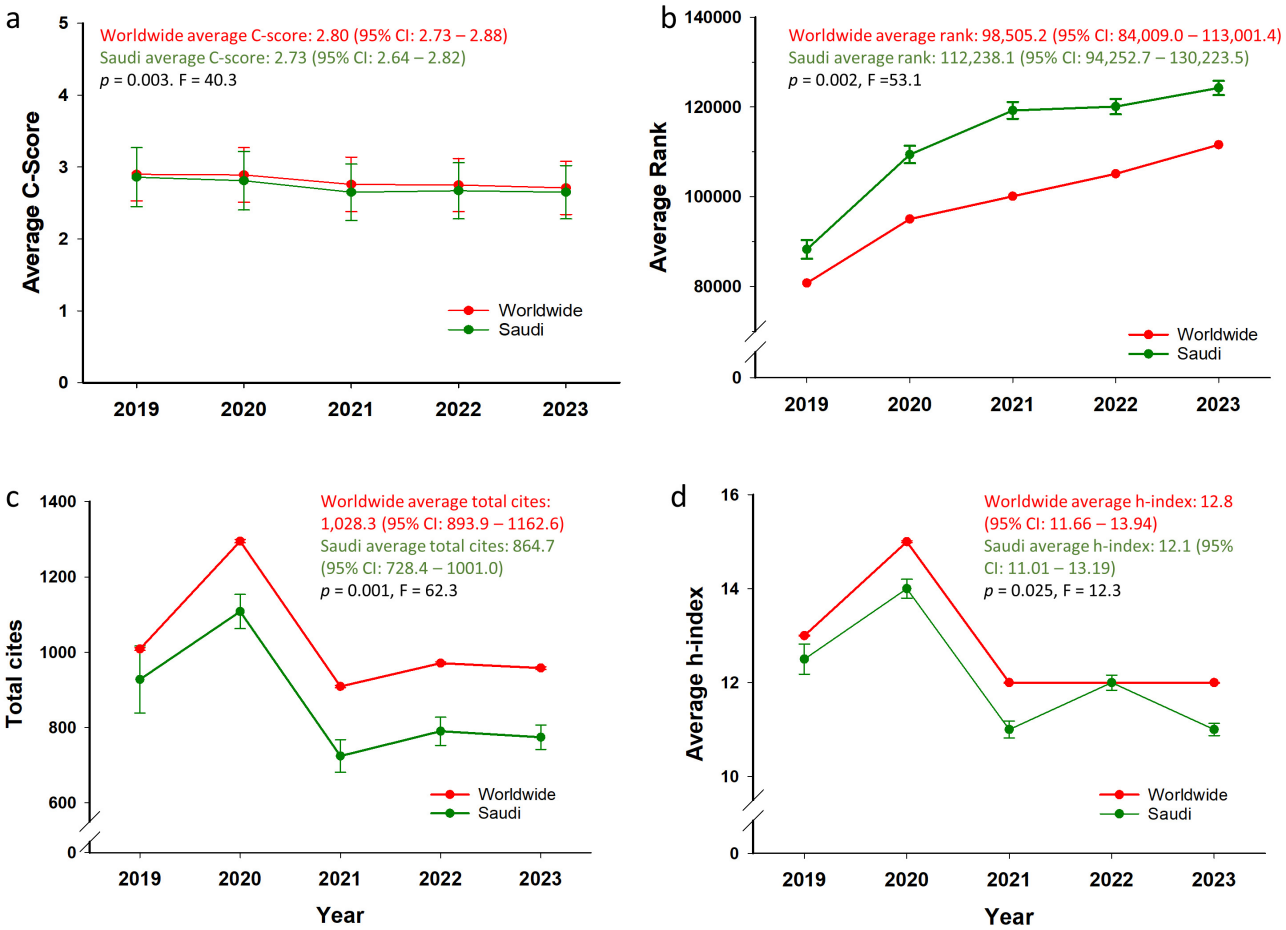
Comparative research performance metrics of Saudi-affiliated and global scientists (2019–2023). **(a–d)** present key bibliometric indicators comparing Saudi-affiliated scientists with their global counterparts from 2019 to 2023. **(a)** Average composite citation scores: Global scientists consistently outperformed Saudi-affiliated scientists, with a statistically significant 5-year difference (2.80 vs. 2.73; *p* = 0.003, *F* = 40.3). The gap narrowed over time, indicating convergence in citation influence. **(b)** Average global ranking: Higher values indicate lower citation standing. Saudi scientists held higher (i.e., less favorable) average ranks than global peers (112,238.1 vs. 98,505.2; *p* = 0.002, *F* = 53.1), though rankings improved steadily, reflecting rising global visibility. **(c)** Total citations: Global scientists accrued significantly more citations (1,028.3 vs. 864.7; *p* = 0.001, *F* = 62.3), with both groups showing similar temporal trends and a peak in 2020. **(d)** Average h-index: Saudi scientists had a significantly lower 5-year average h-index than the global cohort (12.1 vs. 12.8; *p* = 0.025, *F* = 12.3), with both groups peaking in 2020 followed by slight fluctuations. Data source: SUD2% (single-year category). Error bars indicate standard deviations **(a)** or standard errors **(b–d)**.

Across the five-year period from 2019 to 2023, the average c-scores were consistently and significantly higher for global scientists than for Saudi-affiliated scientists (mean of yearly averages: 2.80 vs. 2.73; *p* = 0.003; *F* = 40.3; Figure 1a). Although the year-to-year differences appeared modest, the statistical significance reflected a consistent observable pattern across all 5 years, alongside low within-group variability. When examining citation rankings, Saudi scientists consistently held higher average values (indicating a lower citation impact) than the global cohort across the 5-year period (Figure 1b). The mean annual average rank was 112,238.1 for Saudi-affiliated scientists, which was significantly higher than the global average of 98,505.2 (*p* = 0.002, F =53.1). Although the difference remained statistically significant, Saudi scientists demonstrated clear improvement, with their average rank steadily increasing from 88,225 in 2019 to 124,240 in 2023, reflecting their growing representation in elite citation categories.

The analysis of the total citation counts revealed a consistent disparity between Saudi and global scientists over the 5-year period (Figure 1c). The mean annual average citation count was 1,028.3 for the global cohort and 864.7 for Saudi-affiliated scientists, a statistically significant difference (*p* = 0.001, *F* = 62.3). Although both groups followed similar trajectories, the gap in total citations remained consistent, with a noticeable peak in 2020. These findings underscored a persistent gap in the overall citation volume, even as Saudi researchers continued mirroring global patterns in the annual citation trends.

The h-index trends between 2019 and 2023 also demonstrated that Saudi scientists consistently had slightly lower values than global scientists (Figure 1d). The mean annual average h-index was 12.8 for worldwide researchers and 12.1 for Saudi-affiliated scientists. Although the annual differences were relatively modest, the gap remained statistically significant (*p* = 0.025, *F* = 12.3) due to the consistently lower values Saudi scientists achieved across all 5 years. Following the total citation trends, both groups reached their highest h-index values in 2020, followed by some fluctuations in subsequent years.

### 3.3 Disciplinary representation

**Figure 2:**
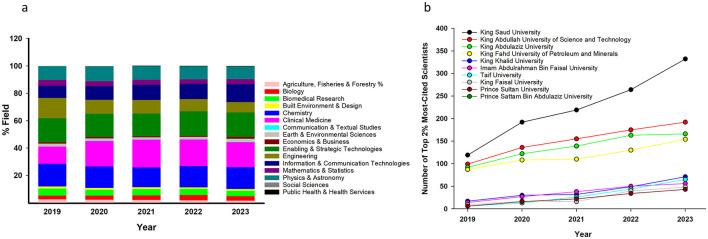
Research field distribution and institutional citation growth of top Saudi scientists (2019–2023). **(a)** and **(b)** summarize disciplinary representation and institutional citation trends among top Saudi-affiliated scientists from 2019 to 2023. **(a)** Disciplinary distribution: Percentage allocation across 22 major scientific fields, based on the Science-Metrix classification. The highest concentrations were in clinical medicine, chemistry, biomedical research, and enabling and strategic technologies. Engineering showed a decline, while mathematics, social sciences, and communication studies remained minimally represented. **(b)** Annual citation growth by institution: Displays cumulative citation contributions over time. King Saud University, King Abdullah University of Science and Technology, and King Abdulaziz University led in overall volume, while Taif University and Prince Sattam Bin Abdulaziz University demonstrated rapid growth, reflecting the nationwide expansion of research excellence.

The disciplinary distribution of Saudi scientists in the SUD2% (Figure 2a) remained broadly stable from 2019 to 2023. The most represented fields across all 5 years were clinical medicine, chemistry, biomedical research, and enabling and strategic technologies, which collectively accounted for a substantial proportion of Saudi-affiliated researchers. Though the relative share of clinical medicine and enabling and strategic technologies remained consistently high, the relative share of engineering demonstrated a gradual decline over time. Meanwhile, mathematics and statistics, communication and textual studies, earth and environmental sciences, and social sciences all demonstrated low representation throughout the study period, suggesting underrepresentation or the limited influence of researchers in these disciplines within the Saudi context.

An institutional breakdown of the Saudi-affiliated scientists listed in the SUD2% single-year dataset (2019–2023) pinpointed King Saud University (KSU) as having the highest representation, with numbers increasing from 119 in 2019 to 332 in 2023, totaling 1,126 cited scientists over the 5-year period (Figure 2b). King Abdullah University of Science and Technology (KAUST) and King Abdulaziz University (KAU) followed closely, showing comparable cumulative totals of 757 and 682 cited scientists, respectively. Both institutions exhibited a steady annual growth and remained among the top contributors of high-impact research throughout the study period. King Fahd University of Petroleum and Minerals also showed consistent growth, reaching 154 listed scientists by 2023 and a total of 589 cited scientists in 5 years.

Beyond these leading institutions, several midsized universities demonstrated significant gains. For example, King Khalid University, Imam Abdulrahman Bin Faisal University, and Taif University (TU) all showed clear upward trajectories, with TU rising from 6 cited scientists in 2019 to 65 in 2023. Similarly, Prince Sattam Bin Abdulaziz University grew from 4 to 54 cited over the same period, reflecting an increasing research engagement.

### 3.4 Gender representation

Of the 5,451 Saudi-affiliated scientists included in the SUD2% dataset from 2019 to 2023, 1,187 entries (21.8%) were excluded from gender classification due to NamSor confidence scores falling below the 85% threshold. Among the remaining 4,264 classified entries, 4,005 (93.9%) were identified as male and 259 (6.1%) were identified as female (Table 2). Across the 5-year period, male scientists' names consistently comprised the vast majority of citations. However, their proportion gradually declined from 95.0% in 2019 to 92.7% in 2023. Conversely, the representation of female names demonstrated a modest upward trend, increasing from 5.0% to 7.3% during the same period (Table 2).

**Table 2:** Gender classification of Saudi-affiliated scientists in Stanford university's top 2% (single-year category).

**Year(s)**	**Male (%)**	**Female (%)**	Unidentified^*^
2019–2023	4,005 (93.9)	259 (6.1)	1,187
2019	381 (95.0)	20 (5.0)	155
2020	631 (94.9)	34 (5.1)	205
2021	765 (94.7)	43 (5.3)	212
2022	984 (93.9)	64 (6.1)	273
2023	1,244 (92.7)	98 (7.3)	342

^*^Uncertain gender classification; confidence score is below 85%.

## 4 Discussion

This study represented the first longitudinal, data-driven assessment of Saudi Arabia's representation in the SUD2% within the single-year category, offering a nuanced perspective on the country's evolving research influence. The use of single-year citation metrics—unlike cumulative, career-long indicators—allowed for a more dynamic evaluation of Saudi Arabia's national research progress, capturing the short-term shifts linked to recent changes in funding, collaboration, and science policy. Such metrics were particularly useful for identifying emerging trends and rising scientific talents, thereby informing evidence-based policy and strategic research planning (Ioannidis et al., 2019, 2016; Al-Marzouqi and Arabi, 2022).

The findings demonstrated a consistent, significant increase in the number of Saudi-affiliated scientists featured in this global research impact benchmark. The number of cited scientists nearly tripled between 2019 and 2023 (Table 1), with institutional participation also expanding. These findings were consistent with a recent analysis of the Top 100 Countries in the SUD2% career-long category between 2017 and 2020, which demonstrated a marked increase in the number of Saudi-affiliated scientists from 65 in 2017 (ranked 39th) to 443 in 2020 (ranked 35th), representing an increase of approximately 580% over the study period (Mujeebu, 2021). This upward trend in both the single-year and career-long categories reinforced the sustained, growing presence of Saudi-affiliated researchers within the global pool of highly cited scientists and aligned with recent national policies aimed at elevating Saudi Arabia's international research profile.

Saudi-affiliated scientists also demonstrated statistically significant gains across multiple citation performance indicators beyond the quantitative increase in representation. Although the average c-score remained modestly below the global average (Figure 1a), the annual differences were both consistent and significant (*p* = 0.003), with Saudi scores converging toward global values as of 2023. This indicated sustained national improvement in research influence despite persistent structural challenges. Similarly, while Saudi scientists held higher (i.e., less favorable) average ranks than the global cohort (Figure 1b), the five-year trend demonstrated the clear upward trajectory in their positioning (*p* = 0.002), further indicating their emerging excellence. Although these results highlight consistent growth, persistent gaps with global averages remain, prompting multiple Vision 2030 initiatives—including reforms in innovation governance, targeted funding of emerging science and technology sectors, and expanded investment in health and biomedical research—aimed at strengthening competitiveness and reducing these disparities (Thalib et al., 2024; Alkhoraif, 2024; Mabkhot and Keong, 2025).

The total citations and h-indices followed comparable patterns. Saudi-affiliated scientists recorded significantly fewer total citations (*p* = 0.001; Figure 1c) and slightly lower h-index values (*p* = 0.025; Figure 1d) than the global average, but these gaps were stable and narrowing. Despite modest annual variations, the statistical significance of these differences reflects their consistency and the relatively low within-group variability. Collectively, these findings support the interpretation that Saudi research is expanding not only in volume but also in international relevance and impact.

The disciplinary representation (Figure 2a) showed a strong concentration in clinical medicine, chemistry, biomedical research, and engineering, which are fields closely aligned with national research and development priorities and Vision 2030 initiatives [Research Development and Innovation Authority (RDIA), 2024]. This finding was consistent with observations from similar national-level studies: (Mujeebu 2021) analyzed country-level contributions to the SUD2% dataset and found that national research agendas often influence field dominance within high-impact lists like SUD2%. Similarly, (Ioannidis et al. 2023) noted that institutional investments and collaboration patterns often drive field-specific momentum.

The institutional analysis further revealed a concentration of high-performing researchers in a few leading universities (Figure 2b). KSU, KAUST, and KAU led by a wide margin, reflecting their established research infrastructures and international collaboration networks. These findings were comparable to those from other countries, such as Greece (Ioannidis et al., 2021), where a few elite institutions were shown to disproportionately drive national citation performance. It is important to note that some Saudi-affiliated scientists were listed under sub-institutional entities like the “College of Science” “College of Pharmacy” and “College of Dentistry” without being explicitly linked to a parent university. Through manual verification using search engines and professional platforms (e.g., LinkedIn, institutional directories), it was confirmed that these individuals were affiliated with KSU. Accordingly, for consistency and accuracy in institutional representation, these entries were consolidated under KSU for the analysis. The rationale for Stanford's disaggregation of these entities was not clearly stated in the original dataset.

Overall, Saudi scientists' steady growth in SUD2% indicated a maturing research ecosystem that is becoming increasingly aligned with global standards. Assuming continued investment and strategic reforms, Saudi Arabia is thus poised to further enhance its scientific influence in the coming decade, particularly in fields with lower current representation (e.g., earth sciences, social sciences).

An 85% confidence threshold was used for gender classification, consistent with the methodology adopted by (Ioannidis et al. 2023), who applied the same criterion using NamSor for their large-scale analysis of scientific authorship. Given that this team also curates SUD2%, this threshold enhanced the methodological alignment of the current study. In the present dataset, 21.8% of entries were classified as being of “unidentified” gender, which was comparable to the 36.1% reported by Ioannidis et al. for global authorship (Ioannidis et al., 2023). In terms of gender distribution, however, the results diverged from the overall global patterns. The global data show that 65.3% of overall authors were male and 34.7% were female, while among top-cited authors 76.2% were male and 23.8% were female (Al-Marzouqi and Arabi, 2022). In contrast, the 5-year average for Saudi-affiliated scientists in this study was 93.9% male and 6.1% female, reflecting a pronounced gender imbalance compared to global benchmarks. However, while the gender gap remained pronounced between 2019 and 2023, the female Saudi-affiliated scientists' representation demonstrated a gradual increase (from 5.0% in 2019 to 7.3% in 2023), suggesting modest progress in gender inclusion within the Saudi research landscape. This trajectory aligns with recent national initiatives aimed at expanding women's participation in research and higher education, particularly under the Vision 2030 framework (Eum, 2019; Saleh and Malibari, 2021; Jawhar et al., 2022).

While this study focused on the Stanford Top 2% dataset derived from Scopus data, future work should extend the analysis to other platforms such as the Web of Science (WoS), the Clarivate Highly Cited Researchers list, and innovation outputs tracked by the World Intellectual Property Organization. Reliance on a single citation-based ranking also has inherent limitations, as the SUD2% may be affected by field-size disparities, self-citation practices, and database coverage. While its standardized methodology enables cross-field comparisons, complementary analyses using alternative frameworks would provide a more balanced assessment of Saudi scientists' impact.

In conclusion, this study has offered the first longitudinal evaluation of Saudi Arabia's position in the SUD2% single-year citation rankings from 2019 to 2023. The findings demonstrated a consistent, statistically significant increase in the number and impact of Saudi-affiliated scientists across multiple bibliometric indicators. Institutional diversity also improved, with more universities contributing to the national research output. Furthermore, the steady increase in female representation suggested the early signs of progress toward inclusivity, though gender disparities persist. These results have underscored the effectiveness of current national research strategies and offer actionable insights for future policy to sustain and expand Saudi Arabia's global science footprint.

## Data Availability

The original contributions presented in the study are included in the article/Supplementary material, further inquiries can be directed to the corresponding author.

## Supplementary material

Supplementary Data 1 Raw data of the Saudi-affiliated scientists in Stanford's top 2 percent most-cited scientists from 2019 to 2023.
